# Comparative results of arthroscopic ankle arthrodesis vs. open arthrodesis in patients with diabetes-associated Charcot Neuro-Arthropathy

**DOI:** 10.1007/s00590-023-03592-0

**Published:** 2023-05-28

**Authors:** Natalia Gutteck, Karl-Stefan Delank, Sebastian Schilde

**Affiliations:** Department of Orthopaedic and Trauma Surgery, Martin-Luther-University Halle-Wittenberg, Ernst-Grube str. 40, 06120 Halle, Germany

**Keywords:** Diabetes-associated Charcot Neuro-Arthropathy, Ankle arthrodesis, Arthroscopic ankle arthrodesis, Minimal-invasive, Foot ulceration, Foot infection, Osteomyelitis

## Abstract

**Background:**

Several studies demonstrated a considerable complication rate for open ankle or TTC arthrodesis in patients with diabetes, revision surgery and ulceration. Extensive approaches in combination with multimorbide patients have been suggested as the rationale behind the increased complication rate.

**Methods:**

Single-centre, prospective case-control study compared arthroscopic vs. open ankle arthrodesis in patients with Charcot Neuro-Arthropathy of the foot. 18 patients with septic Charcot Neuro-Arthropathy Sanders III–IV received an arthroscopic ankle arthrodesis with TSF (Taylor Spatial Frame®) fixation combined with different additional procedures required for infect treatment and hindfoot realignment. The ankle arthrodesis was required for the realignment of the hindfoot in Sanders IV patients, arthritis or in case of infection. 12 patients were treated with open ankle arthrodesis and TSF fixation combined with various additional procedures.

**Results:**

A significant improvement has been shown in radiological data in both groups. A significant lower complication rate has been registered in arthroscopic group. A significant correlation was seen between major complications and therapeutic anticoagulation as well as smoking.

**Conclusion:**

In high-risk patients with diabetes and plantar ulceration excellent results could be demonstrated in arthroscopically performed ankle arthrodesis with midfoot osteotomy using TSF as fixation devise.

## Introduction

The primary aim of the treatment in Charcot Neuro Arthropathy is limb preservation, achieving an ulcer and infection free, plantigrade and shoeable foot. Several studies demonstrated a considerable complication rate for open ankle or TTC arthrodesis in patients with diabetes, revision surgery and ulceration [1, 2]. Extensive approaches in combination with multimorbid patients have been suggested as the rationale behind the increased complication rate [3, 4].

Various approaches and fixation methods have been described for ankle and TCC arthrodesis. In compromised patients with acute or chronic infections, hindfoot malalignment, ulcerations or soft tissue defects, polyneuropathy, peripheral vascular disease the external fixation in Ilizarov or Ilizarov-like device is an established and successful procedure [5–16]. Due to complex deformities, combined procedures with ankle or TTC arthrodesis and midfoot osteotomies are required to restore a plantigrade foot. Regarding the high surgical side complication rate associated with extensive approaches, the arthroscopically and minimal-invasive techniques could be helpful to reduce the surgical trauma.

Encouraging results for arthroscopic ankle arthrodesis with reduced complication rate has been published previously [17–19]. Consequently, arthroscopic ankle and TTC arthrodesis in combination with external fixation could be a promising strategy in compromised patients to reduce the complication rate.

## Materials and methods

### Patients

**Fig. 1:**
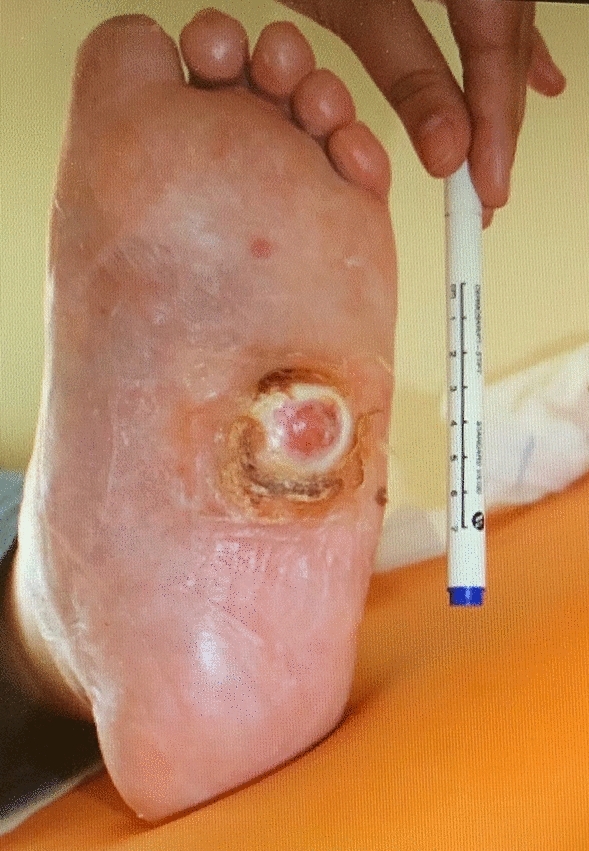
Patient with diabetes associated Charcot Neuro-Arthropathy, plantar ulceration and chronic osteomyelitis

**Fig. 2:**
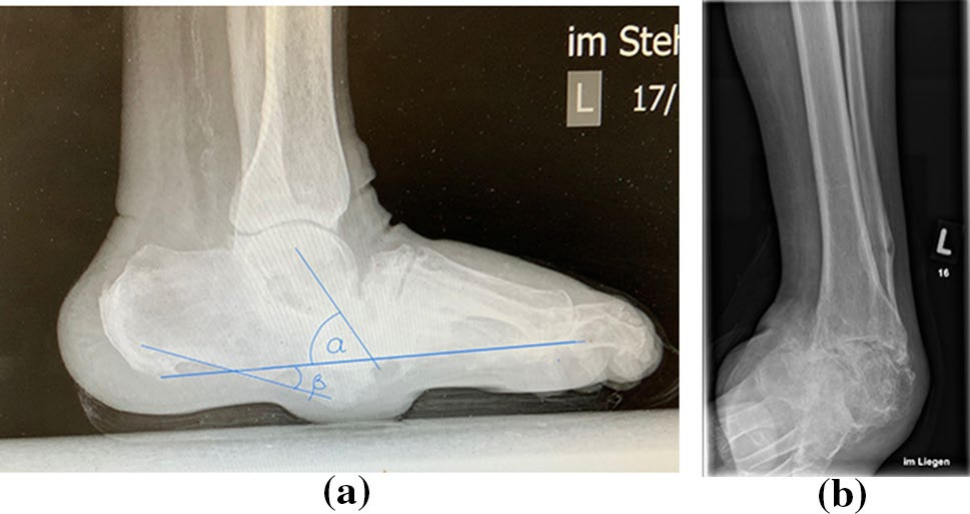
**a** Preoperative lateral weightbearing x-ray with demonstration of the talar declination (*α*) and calcaneal inclination (*β*) angle (artroscopic group). **b** Preoperative lateral weightbearing x-ray (open approach group)

**Fig. 3:**
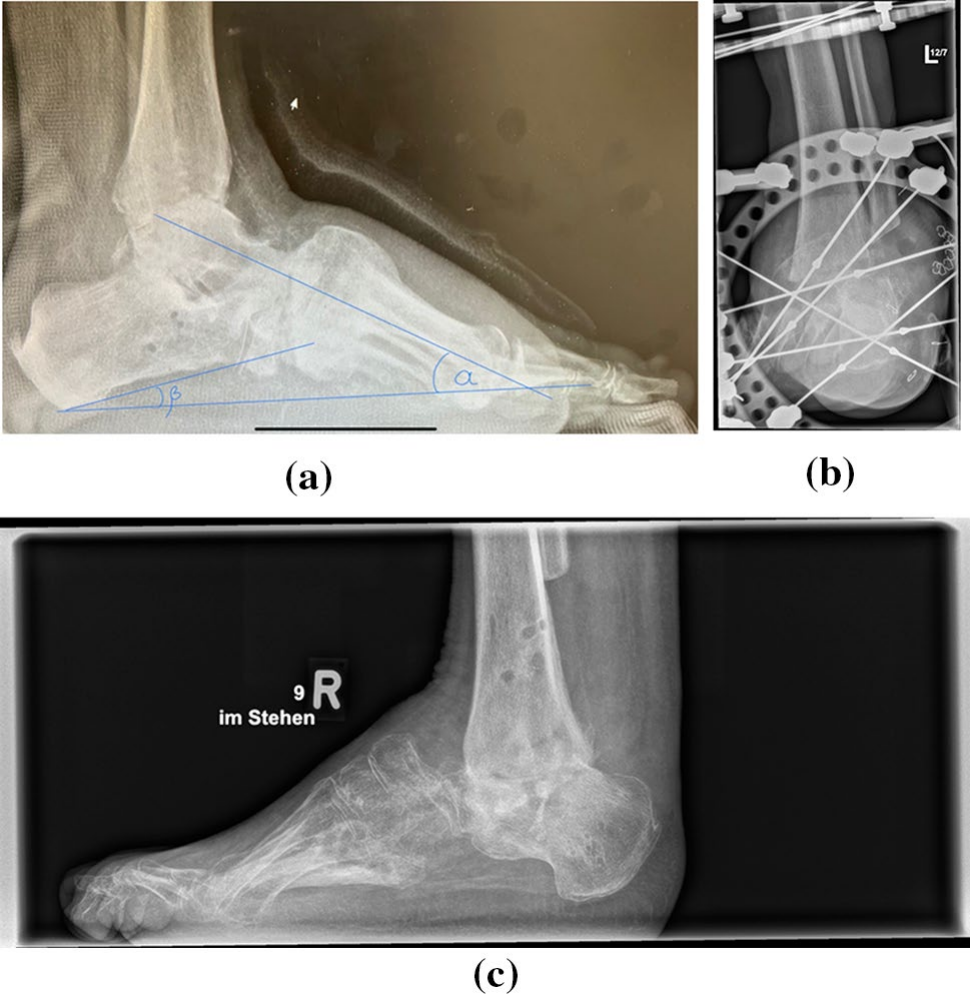
**a** Lateral weightbearing x-ray with demonstration of the talar declination (*α*) and calcaneal inclination (*β*) angle one year postoperatively (arthroscopic group). **b** Lateral weightbearing x-ray with attached TSF (open approach group). **c** Lateral weightbearing x-ray shows fused ankle arthrodesis 3 years postoperatively (open approach group)

Single-centre, prospective case–control study compared arthroscopic vs. open ankle arthrodesis in patients with Charcot Neuro-Arthropathy of the foot. The indication for ankle arthrodesis was Sanders type IV, Sanders III with simultaneously present rigid equinus position of the talus with severe arthrosis of the ankle joint or ankle associated infection. Beginning in 2018, open ankle arthrodesis was gradually replaced by the arthroscopic technique in our clinic. From 2018, 18 patients with Charcot Neuro-Arthropathy of the foot received an arthroscopic ankle arthrodesis with TSF (Taylor Spatial Frame®) fixation combined with different additional procedures required for infect treatment and hindfoot realignment (arthroscopic Group) (Figs. 1, 2, 3). Twelve Patients were treated with open ankle arthrodesis and TSF fixation combined with various additional procedures (open Group). All patients were checked prior to surgery for sufficient blood supply of the affected leg. Interventions were performed, if necessary, by vascular surgery department. The diabetic treatment was checked and adjusted by diabetologist.

### Surgical procedure

In case of acute infection with plantar ulceration and osteomyelitis (Fig. 1) of the midfoot preconditioning surgery with bony debridement or resections in combination with local and systemic antibiotic treatment, negative pressure wound therapy were performed firstly. The Achilles tendon tenotomy was required to resolve the fixed equinus position of the hindfoot and reduce the inner pressure on the soft tissue. In patients with extended bony resection in the midfoot the TSF was applied simultaneously.

After successful wound treatment—which was judged clinically and microbiologically—the wound closure was performed applying local antibiotics (Septopal® Zimmer Biomet). Due to clean clinically appearance under systemic antibiotically treatment, decreasing inflammatory values and good healing under negative pressure wound therapy, a negative microbiological result was not assumed for wound closure. The ankle arthrodesis was performed simultaneously to wound closure.

In open ankle arthrodesis a lateral approach was performed. It allowed especially in cases with extended infection to access the ankle and the subtalar joints as well as the midfoot. The distal fibula was resected 2–3 cm above the ankle joint line. The articular surfaces of the ankle and when required of the subtalar joint were debrided. The subchondral marrow was multiply perforated and the hindfoot realigned and temporary fixed with 2.0 K-wires. After satisfactory position was checked by fluoroscopy, the TSF was installed.

**Fig. 4:**
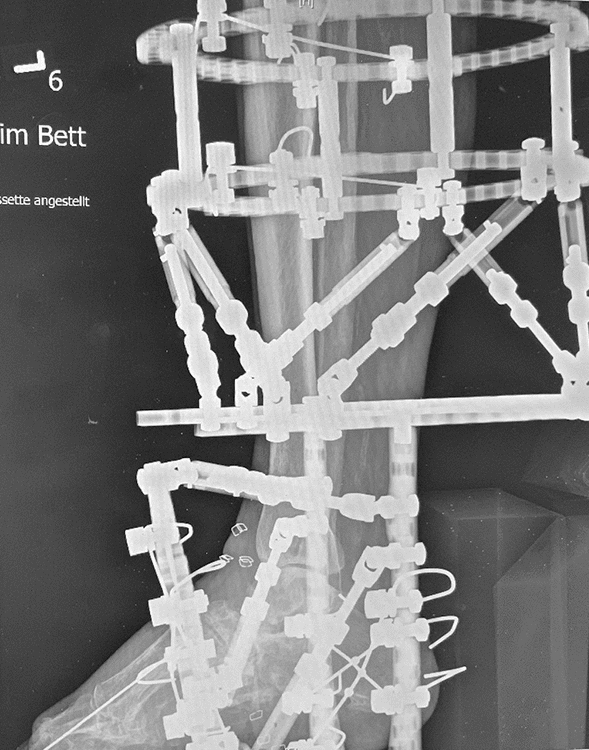
Lateral x-ray shows attached TSF for simultaneous arthrodesis in tibiotalar und subtalar joints

The arthroscopically procedure differed only in the surgical approach. Standard anteromedial and anterolateral portals were applied. Firstly, the intraarticular soft tissue was removed using a shaver. Next, the joint surfaces were debrided using a PoweRasp™ (Arthrex Inc. Naples, Florida, USA) by removing the osteophytes, cartilage and sclerosis (Fig. 4). Due to the alternate use of the portals all parts of the ankle joint could be reached. The preparation of the subtalar joint, if required, was performed minimally invasive through a sinus tarsi approach [20].

**Fig. 5:**
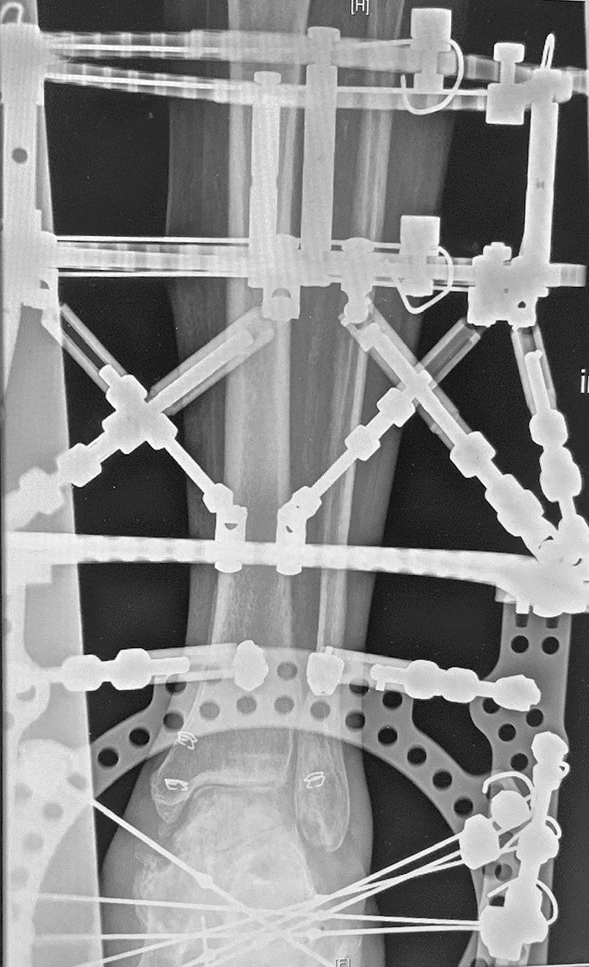
Anteroposterior x-ray view with attached TSF for simultaneous arthrodesis in tibiotalar und subtalar joints

For the TSF apparatus we used two tibial rings, which were attached by 2–3 tensioned wires each. Two “footplates” were rectangular attached to each other for calcaneal fixation with four tensioned olive wires. The proximal “footplate” was connected with tibial rings using rods applying compression to the ankle and subtalar joint. Further ring was used for the forefoot, if bony resections in the midfoot were required. It was fixed with tensioned opposing olive wires passing through the first and fifth metatarsal. The forefoot ring was connected with rods to the “footplate” (Fig. 5).

The postoperative treatment included an average TSF period of 16 (± 5) weeks. The CT scan was performed to judge the fusion. After the TSF was removed, patients were mobilised in long walker with full weight bearing until a custom-made arthrodesis boot was fitted.

### Outcome parameters

Patients were followed up for 1 year and for 3 years in case of complications like nonunions. Occurred complications were registered and were the primary outcome parameter. Major complication was defined as painful non-union, ulcer recurrence, persistent infection, major amputation. Minor complication included non-surgical treated wound problems, wire breaking or wire associated infection, required wire change.

Secondary outcome parameters were radiographic evaluation of the achieved correction, time to bony fusion and the hospitalisation time.

Radiographic evaluation included pre- and 1 year postoperative analysis of the talar declination, calcaneal inclination angle (Figs. 3, 4). The difference between pre and postoperative values was calculated and considered in the statistical analysis. Bony fusion was defined as bone trabecula crossing more than 50% of the former joint [21–24]. Additionally, CT scans were performed at the point of TSF removement in average of four months postoperatively. In cases of delayed bone healing, the CT scans were repeated in average of eightmonths postoperatively.

### Statistical analysis

Statistical analyses were performed using Excel software version 2013 (Microsoft Office, Redmond, WA). Statistical level of significance was set to 5% (*P* ≤ 0.05). No sample size calculation could be performed due to the limited number of patients. Comparison of demographic data between the groups were based on Fisher exact test (nominal variables) and Student *t* test (metric variables). Descriptive comparative analysis of the measured values included mean, standard variation, paired *t* test (pre- and postoperative angle measurements with dependent variables), and Student *t* test. The presence of normal distribution was confirmed using Kolmogorov–Smirnoff test. When testing nominal variables, the Eta coefficient (*η*) was calculated. A one-factor ANOVA was performed for significance testing. Correlations between two continuos parameters were examined using the Pearson correlation coefficient (*r*).

## Results

In total 29 patients with 12 open and 18 arthroscopically ankle arthrodesis was included in to the study. Two patients in the arthroscopic group died after successful treatment due to Covid-19 infection and were excluded from the study. The demographic data and special medical conditions, vascular interventions are demonstrated in Table 1. Additional surgery procedures and in house-stay are summed up in Table 2.

**Table 1:** Demographic data and pre-existing medical conditions

	Open arthrodesis	Arthroscopic arthrodesis
*n*	12	18
Age	64 (± 14)	65 (± 16)
Sex w/m	2/10	1/15
Side (right)	46%	51%
DM Type 2	12	16
DM Type 1	0	2
HgA1c % preop	8,6	8,8
BMI	29 (± 6)	29 (± 8)
ASA classification	2.7 (± 0.5)	2,6 (± 0.5)
Smokers	1	2
Alkohol misuse	3	4
Sanders type 4	6	7
Sanders type 3 + 4	6	9
Peripheral polyneuropathy	12	16
Peripheral arterial occlusive disease	10 (83%)	16 (89%)
Vascular Interventions	5 (42%)	9 (50%)
Arterial hypertension	11 (92%)	16 (89%)
Coronary heart disease	4 (33%)	6 (33%)
Atrial fibrillation	3 (25%)	5 (28%)
COPD	2 (17%)	3 (17%)
Chronic kidney insufficiency	8 (67%)	12 (67%)
Dialysis	4 (33%)	7 (39%)
Cirrhosis liver	0	1 (6%)
Previous surgery	2 (17%)	5 (28%)
Previous Lisfranc Amputation	0	1 (6%)
Present plantar ulceration	9 (75%)	16 (89%)
Previous plantar ulceration	10 (83%)	17 (94%)

*N* number of patients, *w* women, *m* men, *DM* diabetes mellitus, *BMI* body mass index, *ASA* American Society of Anesthesiologists, *COPD* chronic obstructive pulmonary disease

**Table 2:** Additional surgical procedures and in house stay

	Open arthrodesis	Arthroscopic arthrodesis
Extended bony resection midfoot	7 (58.3%)	12 (75%)
5. MT amputation	4 (33.3%)	6 (37.5%)
astragalectomy	2 (16.6%)	0
Operative preconditioning	9 (75%)	13 (81.25%)
Number of preconditioning interventions	2.9 (± 1.7)	2,75 (± 1.8)
In house stay	24 (± 6)	15 (± 4)

*MT* os metatarsale

## Radiographic results

Significant improvement of preoperatively measured angles was registered for all parameters. The talar declination angle (*α*) improved from 38.95° (± 8.05°) preoperatively to 21° (± 6) postoperatively in the arthroscopic group (*p* = 0.00001). The calcaneal declination angle (*β*) improved from 14° (± 7.81) preoperatively to 30.95° (± 6.21) postoperatively (*p* = 0.00001) in the arthroscopic group. The talar declination angle (*α*) improved in the open approach group from 36.83° (± 3.76°) preoperatively to 21.33° (± 3.65) postoperatively (*p* = 0.00001). The calcaneal declination angle (*β*) improved in the open approach group from 15.33° (± 4.29) preoperatively to 28° (± 5.33) postoperatively (*p* = 0.00001). There was no significant difference between groups regarding preoperative and postoperative values and consequently in the correction angle.

## Complications

Two patients (16.6%) in open approach group suffered septic non-union after complex midfoot resection with recurrent ulceration above plantar mid-/hindfoot. They were treated by Pirogoff amputation. Two patients (16.6%) in the open approach Group developed an extended postoperative haematoma due to therapeutically anticoagulation and further two (16.6%) a wound healing problem of the lateral approach to the ankle and had to be revised. The total rate of major complications related to the ankle procedure resulted in 49.8%. Wire breaking or loosening with required revision were registered in 8 patients (66.6%). Two patients required plastic surgery with free flaps and one patient needed a suralis flap (25%).

Arthroscopically treated patients had no major complications on the side of the ankle. One patient developed a postoperative haematoma due to therapeutically anticoagulation after midfoot osteotomy and could be successfully treated by revision surgery (6.25%). One patient had recurrent plantar ulceration and septic non-union after mid-foot osteotomy and was treated by Pirogoff amputation (6.25%). A significant difference between the groups was registered according to complications related to the ankle procedure (*p* = 0.033). Wire breaking or loosening with required revision waere registered in 11 patients (68.75%) with no significant difference between the groups. There was no significant difference according to complications related to midfoot procedures (Table 3).

**Table 3:** Surgery-associated complications

	Open arthrodesis	Arthroscopic arthrodesis
*Major*		
Pirogoff Amputation	1 (8.3%)	1 (6.25%)
Lower leg amputation	1 (8.3)	0
Wound healing problems ankle	4 (33.3%)	0
Wound healing problems midfoot	3 (25%)	2 (12.5%)
Free flap	2 (16.6%)	0
Suralis flap	1 (8.3%)	0
*Minor*		
Asymptomatic non-union	4 (33.3%)	5 (31.25%)
Wire breaking/loosening	8 (66.6%)	11 (68.75%)

**Fig. 6:**
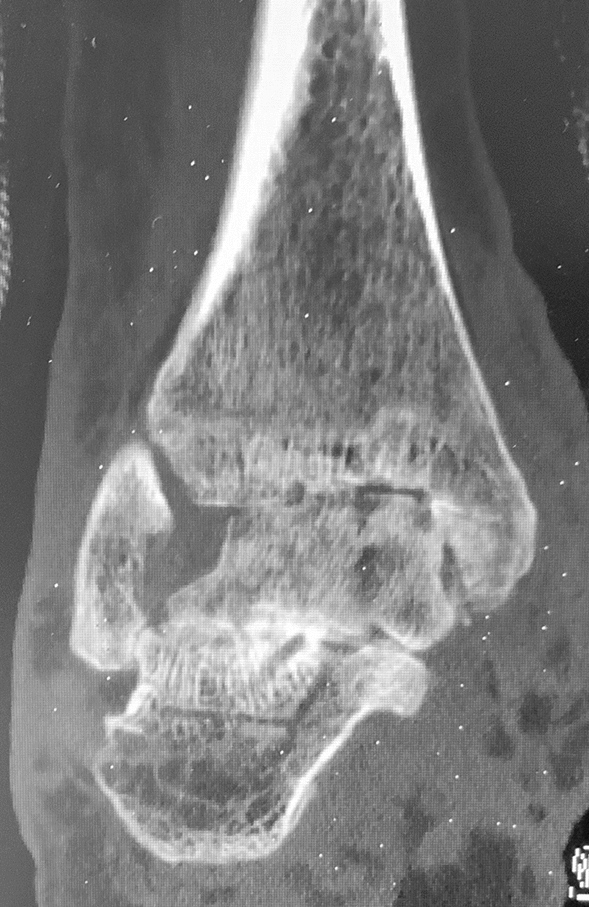
CT scan 14 weeks postoperatively shows fused TTC arthrodesis

**Fig. 7:**
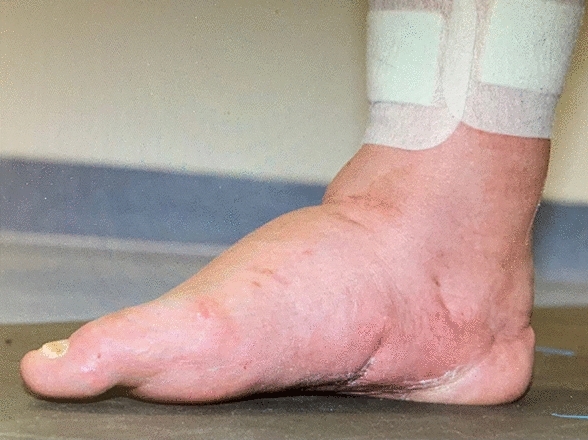
Clinical result of the corrected foot 12 months postoperatively in lateral view

**Fig. 8:**
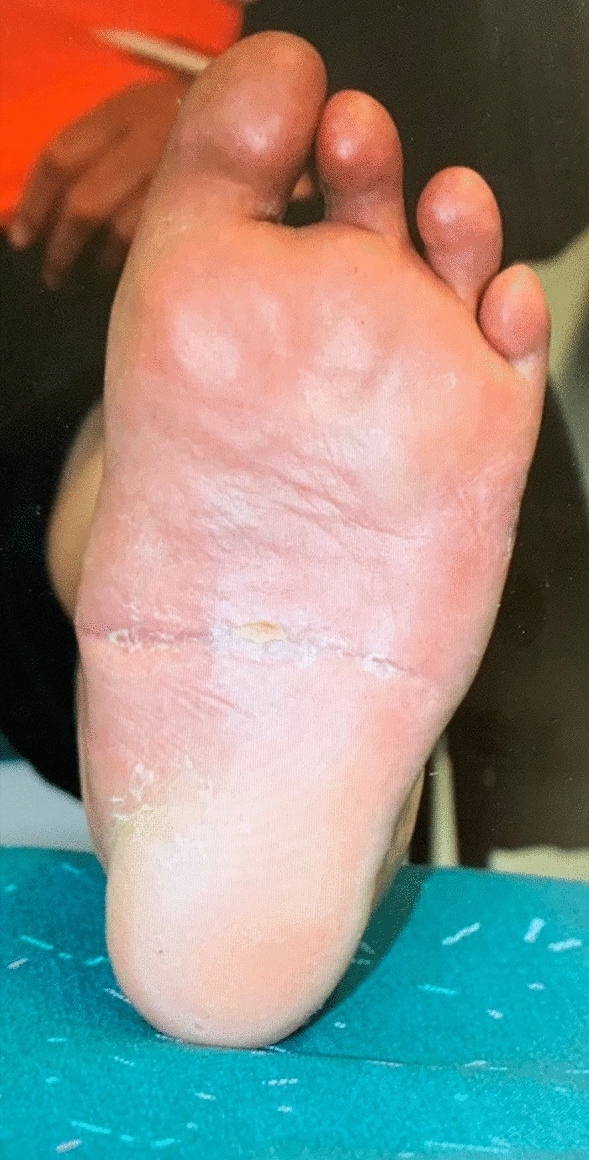
Clinical result of the corrected foot 12 months postoperatively in plantar view

Bony fusion was registered in CT scans in open approach group after 16 ± 7 weeks in 8 patients (66.6%) and in arthroscopic group after 16 ± 5 weeks in 11 patients (68.75%) (Fig. 5). Radiologically 4 patients (33.3%) in open approach group had non-unions. Clinically, they were judged as stable and were non-symptomatically. In CT scans in average 8 months postoperatively, bone healing was registered in further two patients. Five patients (31.35%) in the arthroscopically group had stable, no symptomatic non union in CT scans in average 4 months postoperatively. In CT scans in average 8 month postoperatively 3 patients showed bone healing. No revision surgery due to the pseudarthrosis was required in both groups. Figures 1, 2, 3, 4, 5, 6, 7, 8 demonstrate the follow up of an arthroscopically treated patient).

A significant correlation was seen between major complications and therapeutic anticoagulation (*r* = 0.397; *p* = 0.006) as well as peripheral arterial occlusive disease (*r* = 0.346; *p* = 0.0078) and smoking (*r* = 0.384; *p* = 0.045) regardless of surgical procedure.

Patients with non union were followed clinically for 3 years. No secondary deformation, correction loss or other symptoms like pain or swelling were registered. The documentation of the mobility of the ankle joint in concerned patients presents stiffness in all cases. The mobilisation with full weight bearing was performed in custom-made arthrodesis boot.

## Discussion

Open ankle arthrodesis is an established procedure and might be necessary in treatment of hindfoot pathologies in Charcot neuro-osteoarthropathy, rheumatoid arthritis, posttraumatic and septic arthritis. However, a considerable complication rate up to 56% is reported in a series of studies [1, 24–30]. Additionally, amputation rates of up to 12% were indicated following TTC arthrodesis [1]. Only few studies focus on patients with risk factors like diabetes. Wukich et al. reported of increased complication rate by factor 8 in diabetes [30]. Diabetes was reported as leading risk factor for amputation and surgery side complications [1, 30]. Mendicino et al. compared results after TTC arthrodesis in patients with and without diabetes. An overall complications rate of 80% and major complications of 50% were reported in patients with diabetes [31]. To sum up, diabetes seems to be the leading severe patient specific risk factor for major complications including amputation in extensive surgery of the hindfoot.

Alternative approaches like arthroscopically ankle or TTC arthrodesis are limited to small incisions and reduce dramatically the invasiveness of the surgical treatment. Consequently, we hypothised, the minimally invasive procedure should lead to significantly lower rate of complications in patients with diabetes or other risk factors.

Only few studies compare results of open and arthroscopic ankle arthrodesis in patients with diabetes. The largest cohort was published by Baumbach et al. comparing open TTC arthrodesis in 8 and arthroscopic TTC arthrodesis in 15 patients with diabetes and other risk factors [3, 4]. The stabilisation was performed using a retrograde nail fixation. 50% of their open TTC arthrodesis patients suffered major surgical side complications and one necessitated below-knee amputation. In the arthroscopic group no major surgical side complications were reported. A higher rate of non-union in arthroscopic group was related by the authors to the pre-existing ulceration and low-grade osteomyelitis. The wound debridement, negative-pressure treatment and negative microbiological results prior to arthrodesis did not reduced the risk of non-union. The authors suggested to combine the arthroscopic ankle arthrodesis with external fixation.

Considering the significantly higher rate of non-union and complications in further studies associated with plantar ulceration, results of arthroscopic ankle arthrodesis using TSF fixation were analysed in the present study [32–37]. The authors registered zero ankle side complications in the arthroscopic group compared to 33.2% ankle side complications in the open arthrodesis group. The complication rate of additional midfoot surgery procedures was comparable in both groups. However, the arthroscopic group was significantly shorter hospitalised compared to the open arthrodesis group (*p* = 0.003). The union rate was comparable in both groups. The authors hypothise, that the non-union appear due to deficient bone quality, low grade osteomyelitis, alterated perfusion and other factors related to diabetes. Likely, all patients with radiologically demonstrated pseudarthrosis had clinically a stable hindfoot and were not symptomatically. No revision surgery was needed in both groups regarding the non-union.

The limitation of the study is still the limited size of the groups and the lack of randomisation. Further limitation is the lack of patient reported outcome and the differing indications for ankle arthrodesis. In our experience, the ankle arthrodesis, especially in patients with Sanders III and rigid equinus position of the talus, prevent the secondary deformation and correction loss after corrective midfoot arthrodesis. The results of this investigation will be published separately. Despite the limitations, this study is the largest prospective comparative cohort of arthroscopic ankle arthrodesis in high-risk patients with diabetes.

To sum up, in high-risk patients with diabetes and plantar ulceration excellent results could be demonstrated in arthroscopically performed ankle arthrodesis with midfoot osteotomy using TSF as fixation devise. There was no difference between the groups respective the radiological correction and union results. In our institution, the arthroscopic ankle arthrodesis is a standard procedure in high risk patients especially in patients with diabetes and Charcot arthropathy.
